# PREDICTIVE FACTORS FOR LOSS OF RESPONSE TO ANTI-TNF IN CROHN’S DISEASE

**DOI:** 10.1590/0102-672020200002e1522

**Published:** 2020-11-20

**Authors:** Byanca Rossetti Moreira dos SANTOS, Carlos Henrique Marques dos SANTOS, Vitória Rossetti Moreira dos SANTOS, Claudia Yanina Garcia TORREZ, Daniel PALOMARES-JUNIOR

**Affiliations:** 1 Department of Surgery, Regional Hospital Rosa Pedrossian, Campo Grande, MS, Brazil; 2 Medicine, Anhanguera-Uniderp University, Campo Grande, MS, Brazil

**Keywords:** Crohn disease, Infliximab, Adalimumab, Treatment Failure, Doença de Crohn, Infliximab, Adalimumab, Falha de tratamento

## Abstract

**Background:**

Anti-TNF drugs are a fundamental part of the treatment of Crohn’s disease (CD), so identifying factors related to loss of response is of great importance in clinical practice.

**Aim:**

Identify potential factors related to loss of response to anti-TNF agents in Crohn’s disease patients.

**Methods:**

This is a prospective study of CD patients attending a specialized outpatient clinic using a specific form, including patients with more than one year of follow-up on anti-TNF (Infliximab, Adalimumab or Certolizumab pegol). The information obtained was tabulated and analyzed to identify possible reasons for the loss of response to anti-TNF agents; results were submitted to statistical analysis by chi-square teste considering significant p<0.05.

**Results:**

Sixty-four patients were included, most of them females (56.3%), predominant age group between 26 and 55 years, of whom 25 required optimization, 23 remained in remission with the usual dose and interval, and 16 required switch; most of those who needed switch had hematological problems such as anemia and/or had already undergone surgical treatment for CD.

**Conclusions:**

Anemia and prior CD surgery have been linked to loss of anti-TNF response.

## RESUMO

**Racional:**

Os anti-TNF são parte fundamental no tratamento da doença de Crohn (DC), portanto, identificar os fatores relacionados à perda de resposta tem grande importância na prática clínica.

**Objetivo:**

Identificar potenciais fatores relacionados a perda de resposta aos agentes anti-TNF em pacientes com DC.

**Métodos:**

Trata-se de um estudo prospectivo de pacientes com DC frequentadores de ambulatório especializado por meio de formulário específico, incluindo-se pacientes com mais de um ano de acompanhamento em uso de anti-TNF (Infliximabe, Adalimumabe ou Certolizumabe pegol). As informações obtidas foram tabuladas e analizadas para identificação de eventuais motivos para a perda de resposta aos agentes; os resultados foram submetidos a tratamento estatístico por meio do teste qui-quadrado considerando-se significante p<0,05.

**Resultados:**

Foram incluídos 64 pacientes, sendo a maioria do sexo feminino (56,3%), faixa etária predominante entre 26 e 55 anos, dos quais 25 necessitaram otimização, 23 se mantiveram em remissão com a dose e intervalo habituais, e 16 necessitaram troca de medicamento; a maioria dos que necessitaram troca tinham alterações hematológicas como anemia e/ou já haviam sido submetidos a tratamento cirúrgico pela doença.

**Conclusões:**

Anemia e operação prévia por DC foram relacionados a perda de resposta aos anti-TNF.

## INTRODUCTION

The management of Crohn’s disease (CD) has been profoundly modified by the introduction of biological treatments, in particular by the availability of tumor necrosis factor-a (TNF-a) inhibitors. TNF-a inhibitors were highly effective in CD, reducing hospitalization and surgery rates^3^ ^,^ ^14^. However, despite the availability of such effective agents, CD treatment often remains suboptimal and the disease remain partially uncontrolled^8^. Another important fact of anti-TNF therapy is that it allows mucosal healing to be maintained, which seems to be a good predictor for a decreased need for long-term surgery^8^.

The introduction of anti-TNF in the 1990s changed treatment algorithms and changed the natural history of CD. However, despite the change, patients’ response to this treatment is still difficult to predict when compared to conventional treatments^7^. Up to 30% do not respond (primary non-responders) and nearly half of those who experienced clinical improvement lost their effects during the first year, requiring medication change or optimization (secondary loss of response)^1^.

For the appropriate choice of the best therapeutic approach, it is necessary to consider a number of factors, including the degree of clinical and endoscopic activity of the disease, location, extent, behavior, efficacy of the drug and its potential side effects, prior response to some type of treatment, presence of extraintestinal manifestations or disease-related complications, in addition to the cost-effectiveness issue^4^ ^,^ ^13^. In addition, there are available methods to evaluate possible causes of loss of anti-TNF response, but they are high cost, with little availability, making them unviable^17^, especially in our country. This fact therefore reinforces the need to identify clinical factors that may be useful in identifying patients who are most likely to fail or lose response.

Currently, the goals of treatment are not only symptom control, but mainly sustained control of inflammation through mucosal healing and prevention of irreversible structural lesions and complications (e.g., fistulas, abscesses, stenoses, fibrosis, dysmotility, dysplasia, neoplasia) which, in turn, lead to hospitalization and surgery^8^, so it is necessary to search for new treatment methods as well as to understand why the anti-TNF response fails aiming decrease complications and improve patients quality of life. Considering that there are few agents available to treat a chronic, progressive and debilitating disease such as CD, identifying possible reasons for drug failure and adapting the available drugs to the profile of each patient is fundamental.

Thus, the aim of the present research was to identify possible factors related to loss of response to anti-TNF agents in Crohn’s disease patients.

## METHOD

This study was approved by the Committee on Ethics in Research of Rosa Pedrossian Regional Hospital, under protocol number 038/2018. All patients were informed about the research, the purpose of data collection and confidentiality with the information. Those who agreed to participate read and signed an informed consent form.

This is a prospective study with analysis of information from patients with CD under follow-up at the outpatient clinic for inflammatory bowel disease. The analysis was performed using a form containing demographic and disease-related data (gender, age, comorbidities, surgery due to CD, medications used, evolution, complications, laboratory abnormalities, disease activity, serological markers, extraintestinal manifestations and complications) in addition to the current assessment of disease activity using the Harvey- Bradshaw index.

All patients who underwent regular treatment with any anti-TNF agent (Infliximab, Adalimumab or Certolizumab pegol) for the disease for at least one year from January 2017 to January 2019 were included. The ones who did not receive regular treatment for at least one year and those whose records were incomplete and unable to extract essential information were excluded.

According to the data found regarding drug use, patients were divided into three groups: 1) group 1 (switch): patients that needed anti-TNF switch; 2) group 2 (adapted): patients adapted to medication, no switch or optimization; 3) group 3 (optimization): patients that needed anti-TNF dose or interval adjustment

After the patients were distributed in the groups, all collected variables were crossed in order to obtain those that could justify the loss of response to anti-TNF.

### Statistical analysis

The results were placed in a spreadsheet for later evaluation and subjected to statistical analysis by the SPSS version 17 program, using the chi-square test, with a significance level of p <0.05.

## RESULTS

Sixty-four patients were included, of which 43.8% were male and 56.2% female. The predominant age range among these patients was from 26 to 55 years (Table 1). Of the 64 patients analyzed, 16 required medication switch, 23 remained adapted to the medication and 25 required dose or interval optimization. No statistically significant differences were observed between groups regarding gender, age and comorbidities (Table 1).

**TABLE 1:** Main characteristics of the patients

	Group 1	Group 2	Group 3	Total	p			
	n	%	n	%	n	%	n	
Gender								
Male	7	43.8	8	34.8	13	52.0	28	>0.05
Female	9	56.2	15	65.2	12	48.0	36	
Age interval								
16- 25	3	18.8	3	13.0	2	8.0	8	>0.05
26- 35	3	18.8	4	17.4	5	20.0	12	
36- 45	6	37.5	4	17.4	7	28.0	17	
46- 55	4	25.0	4	17.4	5	20.0	13	
>55	-	-	8	34.8	6	24.0	14	
Comorbidities								
Systemic arterial hypertension	2	66.6	3	60.0	5	62.5	10	>0.05
Diabetes mellitus	0	0	1	20.0	0	0	1	
Lactose intolerance	0	0	1	20.0	1	12.5	2	
Depression	1	33.3	0	0	2	25.0	3	

Table 2 shows that in the group 1 (switch), 50% had anemia and no platelet increase, while in the other groups this percentage was 8.7% and 12% (p <0.05).

**TABLE 2:** Hematological changes observed in the groups

		Not rated	Anemia yes, thrombocytosis yes	Anemia yes, thrombocytosis no	Anemia no, thrombocytosis no	Total	p
Group 1	Score %	0 0%	4 25%	8 50%	4 25%	16 100%	p<0.003
Group 2	Score %	2 8,7%	2 8,7%	2 8,7%	17 73,9%	23 100%	p<0.003
Group 3	Score %	5 20%	2 8%	3 12%	15 60%	25 100%	p<0.003
Total	Score %	7 10%	8 12.5%	13 20.3%	36 56.3%	24 100%	p<0.003

Another fact evidenced by the analysis was that the group that needed anti-TNF switch had a higher percentage of surgical procedures due to complications of the disease. In group 1, 62.5% of patients underwent surgery, while in group 2 they were 39.1% and in group 3, 24% (p<0.05).

**TABLE 3:** Need for prior surgery due to Crohn›s disease.

		Yes	No	Total	p
Group 1	Score %	10 65.2%	6 37.5%	16 100%	p<0.048
Group 2	Score %	9 39.1%	14 60.9%	23 100%	p<0.048
Group 3	Score %	6 24%	19 76.0%	25 100%	p<0.048
Total	Score %	25 39.1%	39 60.9%	64 100%	p<0.048

Applying the Harvey-Bradshaw index, when groups 1 and 3 were analyzed together, i.e., those requiring medication change and those needing dose or interval optimization, it was observed that they had higher disease active than those in group 2, adapted to the medication. The clinical remission in the medication-adapted group was then 1.47 times higher than in the non-adapted group (p<0.05, Table 4).

**TABLE 4:** Crohn›s disease activity in patients by group according to Harvey-Bradshaw Index*

		Inactive disease	Active disease	Total	p
Groups 1+3	Score %	7 18.4%	31 81.6%	38 100%	p<0.025
Group 2	Score %	10 45.5%	12 54.5%	22 100%	p<0.025
Total	Score %	17 28.3%	43 71.1%	60 100%	p<0.025

*According to Harvey-Bradshaw Index <5 points=remission; 5 points=active disease

The other information collected related to the disease characteristics of each patient belonging to the studied groups did not present statistically significant differences and are presented in Table 5.

**TABLE 5:** Data related to the disease characteristics of the patients in the studied groups

	Group 1	Group 2	Group 3	p
Number of patients	16	23	25	>0.05
Association with immunosuppressant	15	17	21	>0.05
First anti-TNF used				
Infliximab	9	14	13	>0.05
Adalimumab	7	9	12	
Second anti-TNF used				
Infliximab	6	-	-	>0.05
Adalimumab	7	-	-	
Certolizumab	3	-	-	
Disease presentation at diagnosis (HBI)				
Mild	3	10	6	>0.05
Moderate	8	10	14	
Severe	5	3	5	
Current disease activity				
Inactive	12	23	23	>0.05
Active	4	0	2	
Weight loss >5% at diagnosis				
Yes	12	9	11	>0.05
No	4	13	13	
Isolated perianal disease				
Yes	1	5	2	>0.05
No	15	18	23	
High serological markers				
CRP	5	5	5	>0.05
ESR	1	1	2	
CRP + ESR	5	0	1	
Elevated calprotectin	2	5	7	>0.05
Extraintestinal manifestations	2	4	5	>0.05
Disease complications				
Toxic megacolon	1	0	1	>0.05
Obstruction	2	3	4	
Bleeding	3	0	0	
Perfuration	3	0	0	

HBI=Harvey-Bradshaw Index; CRP=C-reactive protein; ESR=erythrocyte sedimentation rate

## DISCUSSION

One of the signs that the disease may not be well controlled is the hematological changes. Several studies agree that anemia is associated with increased disease activity, use of health services, mortality and poor quality of life^2^. These data are in agreement with the present study, which showed that patients requiring anti-TNF switch had a higher rate of anemia than other patients with CD.

In a study^16^ in which quality of life was assessed by the Inflammatory Bowel Disease Questionnaire (IBDQ), patients with anemia had lower scores compared with non-anemic IBD patients after adjusting for disease activity. In this study, failure to identify cause of anemia (i.e., lack of ferritin and CRP results after documented anemia) and failure to perform annual anemia assessments generally provide evidence of a gap between guidelines and clinical practice. Adherence to anemia screening guidelines has the potential to impact the clinical and social life of patients with IBD. Of course, it is not enough to recognize and treat anemia as the guidelines recommend, but rather to act promptly in recognizing its cause, which as demonstrated here may be disease activity due to failure or loss of response to anti-TNF.

In fact, the CHARM^5^ randomized study reported a significant reduction in the risk of hospitalization and surgery for moderate to severe CD in case of adalimumab maintenance compared to placebo. Similarly, in the ACCENT II^15^ study, infliximab maintenance was associated with a drop of more than 50% hospitalizations and surgeries for patients with fistulizing Crohn’s disease. This trend was confirmed by a recent population-based study in Sweden^9^, which found that the proportion of patients undergoing CD-related surgery within five years of diagnosis decreased from 65.8% to 34.6% between 1963 and 2005. However, few studies^14^ bring data regarding surgical complications in patients who were already using anti-TNF and yet needed to change or optimize the medication.

In the present study, it can be evidenced that those patients who required medication switch had a higher rate of evolution to surgery compared to the other groups. Already the groups adapted to the medication or just needing to optimize it, follow the general statistics presented in the large studies as ACCENT II^15^. A study published in the European Crohn’s and Colitis Organization (ECCO)^10^ shows that the rate of hospitalizations and complications increases relatively in patients needing medication change, as it also coincides with our data. In the same study^10^, it was shown that medication dose optimization was required in 34.9% of patients with first-line treatment and 37.1% in patients with other treatment lines, a result very similar to the present study in which 39% of patients required dose or interval optimization of the medication.

In the original article published by Mege et al.^12^, some preoperative risk factors that may affect the effectiveness of postoperative anti-TNFa antibody treatment in CD patients were analyzed: younger age at diagnosis, antibody treatment anti-TNFa before surgery and residual inflammation outside the surgical site. Although this was not the focus of our research, this data also seems to reinforce what has been shown here.

The main reason for the need for surgery, despite anti-TNFa antibody treatment, appears to be primary ineffectiveness or secondary loss of response to the agent. Most cases with secondary loss of response are known to be associated with the development of anti-drug antibodies^6^. Antibodies developed before surgery are likely to work against any anti-TNFa antibody treatment administered after surgery. Therefore, those who initially responded to anti-TNFa agents, but subsequently lost response should recur despite postoperative anti-TNFa antibody treatment.

Regarding the clinical remission achieved by the use of anti-TNF, studies show^11^ that in patients in whom remission was not achieved in the first year of therapy, after an average treatment period of three years of medication, the adequate clinical response is not reached in half of these patients. This would justify the fact that the groups 1 and 3 not adapted to the medication present the HBI corresponding to the active disease.

The need for early surgery after initiation of anti-TNFa antibody treatment may not indicate primary agent ineffectiveness, but insufficient efficacy due to the presence of intestinal complications, as the presence of intestinal complications is known as one of the risk factors for ineffectiveness of anti-TNFa agents^11^. Therefore, in these patients, removal of intestinal complications by surgery may reconstitute the efficacy of anti-TNFa agents. Thus, early surgical intervention after primary non-responsiveness or surgical intervention before the initial initiation of anti-TNFa agents may be effective for patients with intestinal lesions applicable to surgery.

Although there is some evidence of a worse prognosis of CD, which could also lead to a lower response to anti-TNF agents, such as early age at onset of the disease, significant weight loss at diagnosis, use of corticosteroids at diagnosis, and extension of the inflamed segment, in the present research these factors did not contribute to the therapeutic failure, but it is necessary to consider the small number of patients studied and the fact that it is a single center, these being the main limiting factors of the present research. However, considering the small amount of research for this purpose in our country, we understand that it contributes to the national medical literature by presenting results from a referral center in IBD.

## CONCLUSION

Anemia and prior CD surgery have been linked to loss of anti-TNF response. 
